# Giant Villous Adenoma of the Rectum With Prolapse: Case Report

**DOI:** 10.7759/cureus.50079

**Published:** 2023-12-06

**Authors:** Iurii Munteanu, Munteanu Mihaela, Silvia Popescu, Iulian M Slavu, Anca Oprescu Macovei, Daniel Cochior

**Affiliations:** 1 General Surgery, Lotus Hospital, Ploiesti, ROU; 2 Radiology, CF2 Clinical Hospital, Bucharest, ROU; 3 Surgery, CF2 Clinical Hospital, Bucharest, ROU; 4 Anatomy, Carol Davila University of Medicine and Pharmacy, Bucharest, ROU; 5 Gastroenterology, Agrippa Ionescu Hospital, Bucharest, ROU; 6 General Surgery, Monza Hospital, Bucharest, ROU

**Keywords:** proctology, emergency gastroenterology and endoscopy, villous tumor, inferior digestive hemorrhage, giant villous adenoma

## Abstract

Colorectal polyps, frequently adenomas, are common in older adults, with villous adenomas being a notable subset due to their potential for significant malignancy risk. This case report highlights a rare instance of a giant villous adenoma in a 79-year-old female patient, challenging in both diagnosis and treatment. The patient, with a history of untreated essential arterial hypertension, was hospitalized for severe anemia following a massive rectal hemorrhage. An irreducible, prolapsed rectal mass was evident upon examination, and further investigations, including rectoscopy and abdominopelvic computed tomography scan, confirmed a villous adenoma with severe dysplasia. Given the tumor's substantial size, circumferential nature, and proximity to the dentate line, an abdominoperineal resection using the Miles technique was performed. The histopathological examination post-surgery confirmed the presence of a villous adenoma with high-grade epithelial neoplasia and localized areas of well-differentiated tubular adenocarcinoma. This case underscores the diagnostic and management complexities of large villous adenomas, emphasizing the need for meticulous surgical decision-making to ensure oncological safety and patient welfare, particularly when conservative resection may be inadequate.

## Introduction

Colorectal polyps, which are adenomas in approximately two-thirds of cases, occur in 25% to 30% of individuals over the age of 50. Villous adenomas, comprising approximately 10% of colorectal adenomas, are rare premalignant lesions typically larger than 5 cm and carry a malignancy risk of up to 40% [1,2]. They often present without symptoms. Common clinical manifestations include excessive mucus secretion, diarrhea, altered bowel habits, difficulty in defecation, or iron deficiency anemia. In rare cases, substantial growth leads to hypersecretory complications such as persistent diarrhea, electrolyte imbalances, and renal failure, a condition known as McKittrick-Wheelock syndrome [3,4]. Giant villous adenomas and anorectal prolapse co-occurrences are rare [5]. This report describes a patient hospitalized for severe anemic syndrome due to a hemorrhaging giant villous anorectal tumor with partial prolapse, exacerbated during exercise or defecation.

## Case presentation

We report the case of a 79-year-old female patient from a rural area with untreated essential arterial hypertension. She was admitted for severe anemia following a massive rectal hemorrhage. The patient's history revealed a year-long tumor progression, initially exhibiting occasional, reducible prolapses during defecation, later progressing to permanent, irreducible prolapse. In the month before admission, she experienced several significant rectal hemorrhages, prompting medical consultation. She reported needing bowel movements to alleviate discomfort from the prolapsed tumor.

**Figure 1:**
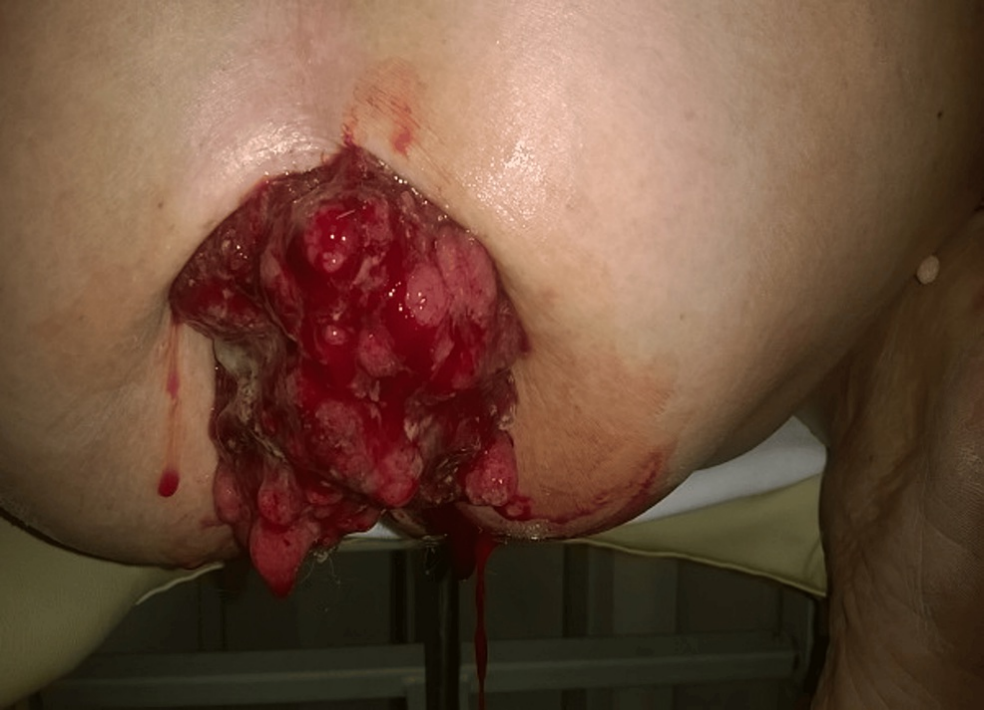
Prolapsed rectal tumor: clinical presentation A photograph showing the prolapsed rectal tumor as observed during physical examination.

The general physical examination showed a pale, dehydrated patient with minimal adipose tissue and body mass index within the reference range, thoracic kyphosis, slightly increased cardiac dullness, normal blood pressure, and regular heart rhythm (Table 1). Hematological tests indicated anemia due to blood loss, with decreased mean corpuscular volume, hematocrit, red blood cell count, and mean corpuscular hemoglobin concentration. CEA (carcino-embryonic antigen) tumor marker was obtained and was in normal ranges. Biochemical analysis revealed low potassium and chloride levels, likely due to recurrent emesis, before presenting to the hospital. Also, she did not eat well due to abdominal pain and the prolapse of the adenoma. These two factors, combined, most probably led to the low level of potassium. The imbalance was corrected before surgery. The local physical examination identified an irreducible, malodorous, prolapsed rectal mass. It was ovoid, bulky, erythematous, with an irregular surface, soft to palpation, measuring 10 cm × 6 cm, and associated with significant hemorrhage (Figure 1).

**Table 1:** Clinical and paraclinical evaluation of the patient at admission BPM: beats per minute; MCV: mean corpuscular volume; MCHC: mean corpuscular hemoglobin concentration; MCH: mean corpuscular hemoglobin.

Investigation	Patient Value	Reference Range
Blood pressure	130/87 mmHg	<120/<80 mmHg
Body mass index	18.6 kg/m^2^	18.5–24.9 kg/m^2^
Heart rate	67 BPM	60–100 BPM
Hematocrit	25.5%	41–50%
Hemoglobin	7.5 mg/dL	12–16 mg/dL
MCV	74 fL	80–100 fL
MCHC	29.4 g/dL	32–36 g/dL
MCH	22 pg/cell	27–31 pg/cell
Red blood cells	3.41 cells/µL	4.2–-5 cells/µL
Potassium	2.8 mEq/L	3.5–5.2 mEq/L
Chloride	95 mEq/L	96–106 mEq/L

On admission, the patient reported malaise following an incomplete tumor prolapse and abundant hemorrhage during defecation. The repeat general physical examination findings were consistent with the initial presentation. Hematological and biochemical tests yielded similar results. The findings of the local physical examination were also consistent with the earlier description.

A thoracic radiograph revealed no progressive pulmonary lesions. However, it showed signs of healed rib fractures (right anterior second and posterior seventh costal arches), enhanced peribronchovascular interstitium, a widened and tortuous descending aorta with calcifications, and a bulging lower left cardiac border. Rectoscopy identified the tumor's inferior limit near the dentate line, extending approximately 10 cm above it. Preoperative biopsies confirmed a villous adenoma with multiple areas of severe dysplasia. A full-length colonoscopy was not attempted before the surgery as the tumor was fragile and brittle and bled easily.

**Figure 2:**
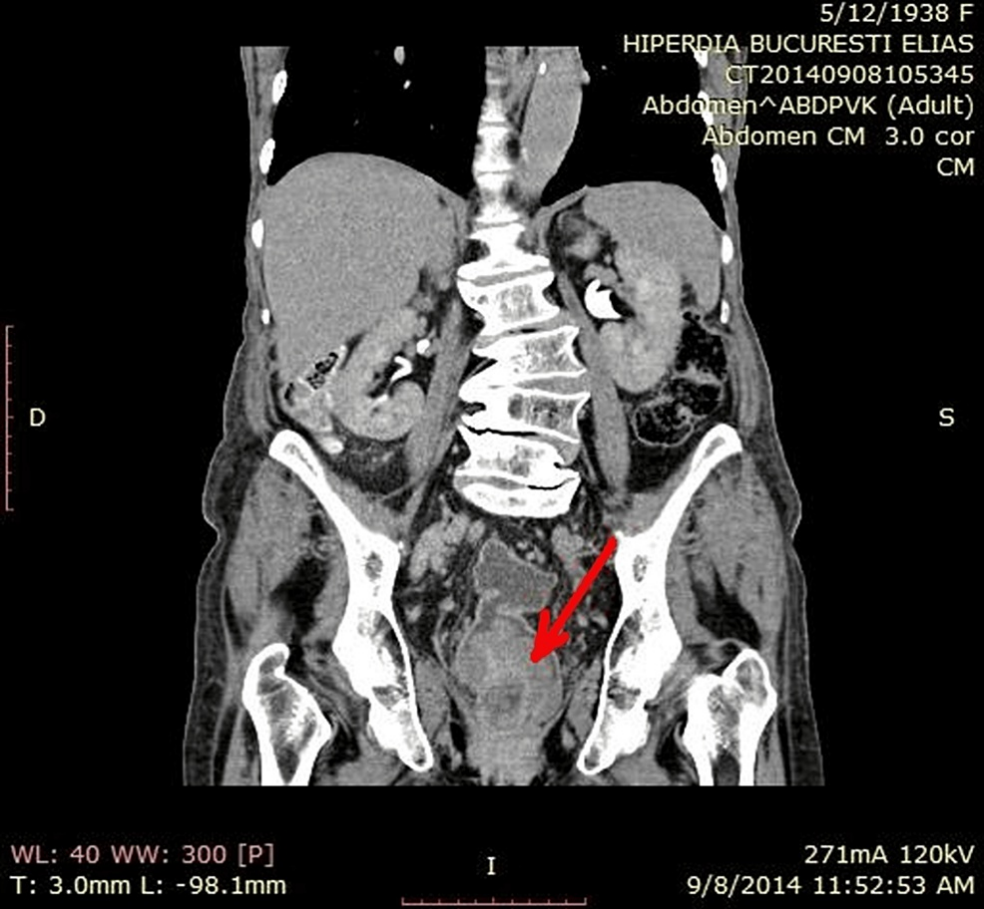
Computed tomography scan of the anal-rectosigmoid mass Coronal view showing the anal-rectosigmoid mass measuring 6.8 cm × 5.3 cm, with invasion into perirectal structures and multiple local-regional adenopathies. Degenerative disc and vertebral changes are highlighted with arrows indicating the tumor location.

Abdominal ultrasound showed an enlarged rectal ampulla with thickened walls and inhomogeneous content. The thorax and abdominopelvic computed tomography (CT) scans suggested malignancy in the lower rectal mass. It revealed distention of the rectal ampulla and rectosigmoid junction, a highly vascularized vegetant tumor with a wide base measuring 6.8 cm × 5.3 cm, extending 8.2 cm toward the anal canal, and infiltrating perirectal adipose tissue and right perirectal fascia. We noted bilateral adenopathies, with the largest measuring 3 cm (left external iliac). Degenerative disc and vertebral changes were observed without osteolytic or osteoblastic lesions (Figure 2). Besides the rectal tumor, no other lesions were observed.

**Figure 3:**
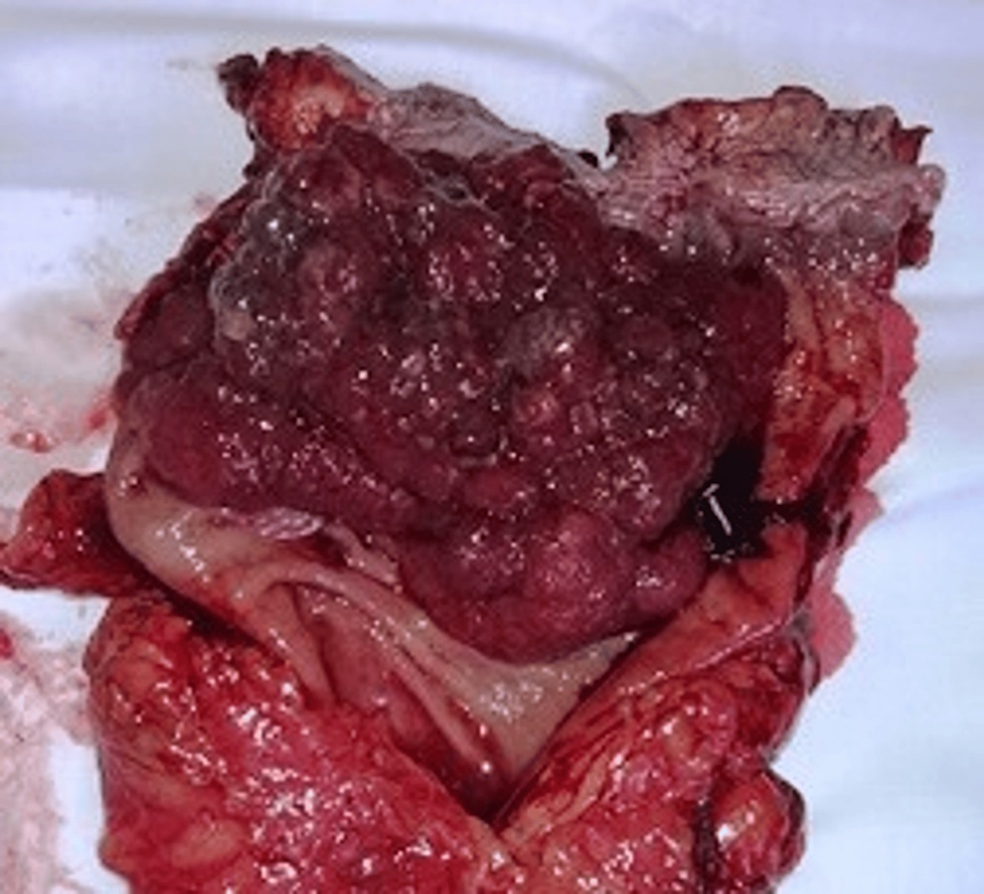
Resected specimen of the rectum with intraluminal vegetative tumor A photograph of the longitudinally cut specimen of the rectum, displaying the intraluminal vegetative tumor.

Taking into consideration the differential diagnosis of invasive adenocarcinoma of the rectum and the CT characteristics, the clinical TNM staging was established as T4aN2bM0. The patient underwent surgery on day 2 after admission to correct the anemia, followed by an abdominoperineal resection of the rectum using the Miles technique. The removed specimen was sent for a histopathological examination (Figure 3). The patient's postoperative evolution had been uneventful; she stayed in the hospital for five nights and six days and was discharged after stool passed through the colostomy. She came for a clinical evaluation 14 days after discharge, 6 months, and 1 year. At the six-month reevaluation, a colonoscopy was done through the colostomy, and no relapse was observed. A thorax, abdomen, and pelvis CT with contrast obtained at the one-year evaluation showed no signs of tumor relapse. CEA showed normal values. After one year, the patient did not return for further evaluation. Further evaluation would have included a yearly CT of the thorax, pelvis, and abdomen with contrast and a colonoscopy for up to five years after surgery.

Macroscopic examination of the 25-cm resected specimen showed that its lower end obscured the pectinate line, extending proximally for 10 cm. The tumor was a large, circumferential, erythemato-violaceous, vegetative mass of soft consistency. Small, adherent blood clots covered its irregular surface. Twenty tissue fragments from different areas were selected for further histopathological examination.

**Figure 4:**
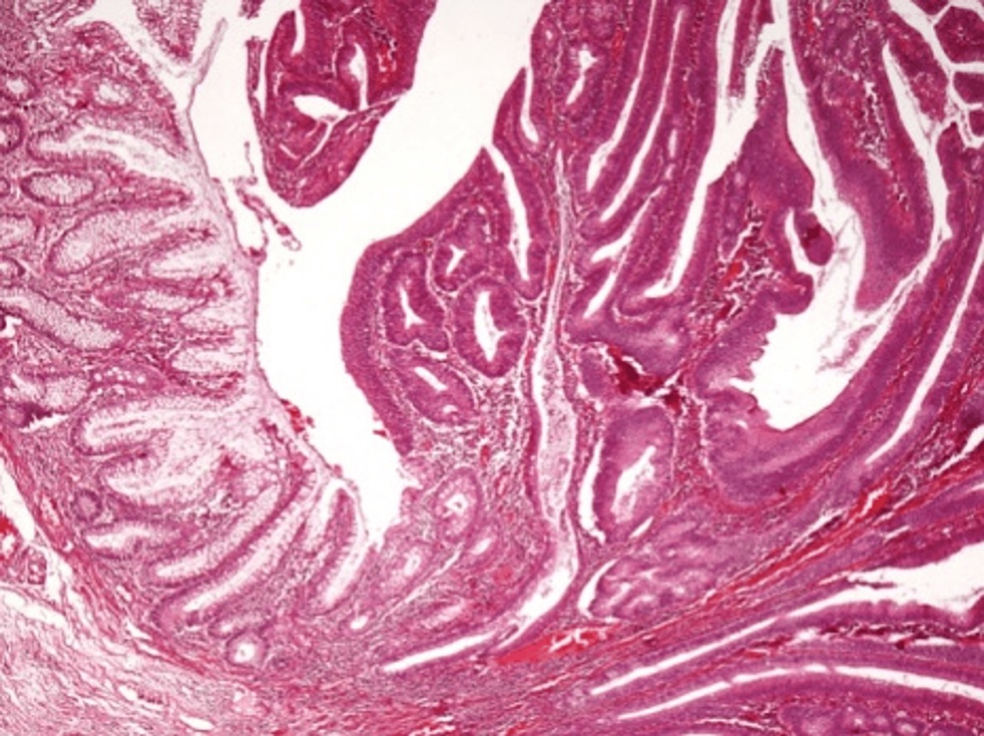
Microscopic evaluation of the villous adenoma A microscopic view of the villous adenoma, detailing the general histopathological features.

**Figure 5:**
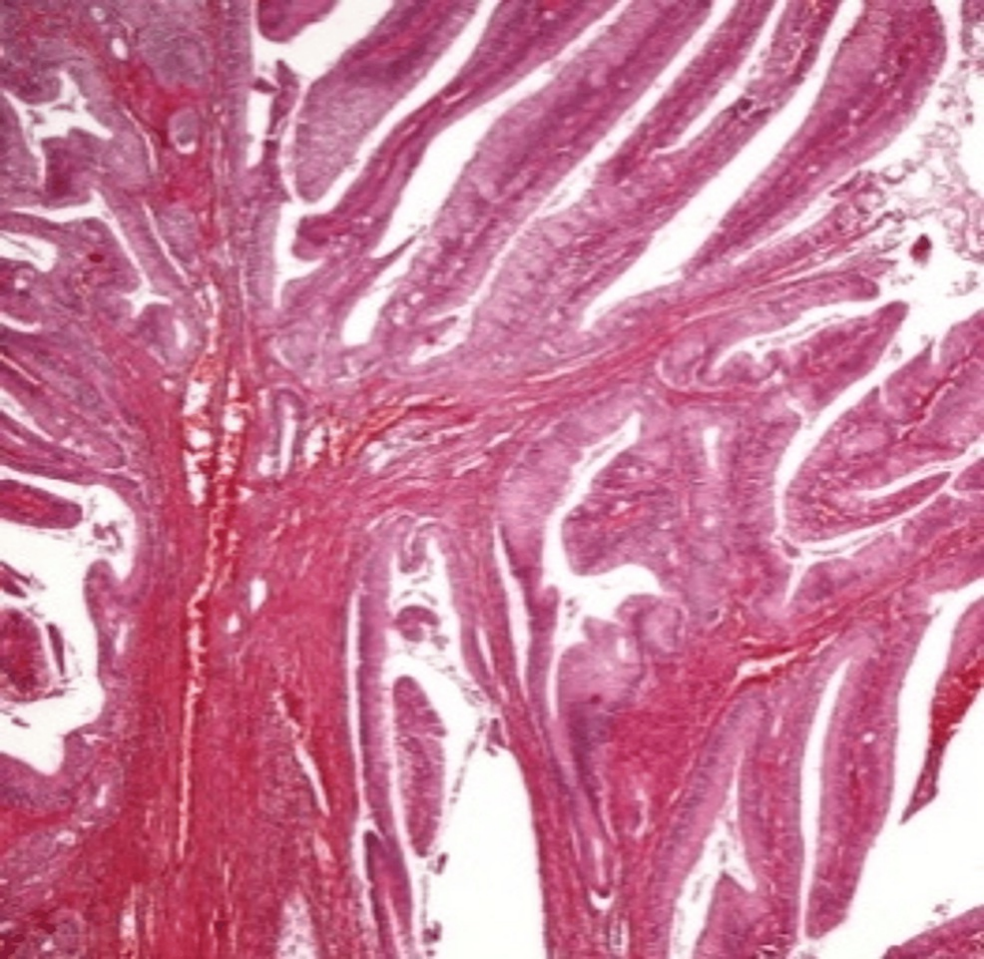
Histopathology: fibrous septa with tumor epithelium A microscopic view showing delicate fibrous septa covered by tumor epithelium without significant nuclear pleomorphism (hematoxylin and eosin stain, 100× magnification).

**Figure 6:**
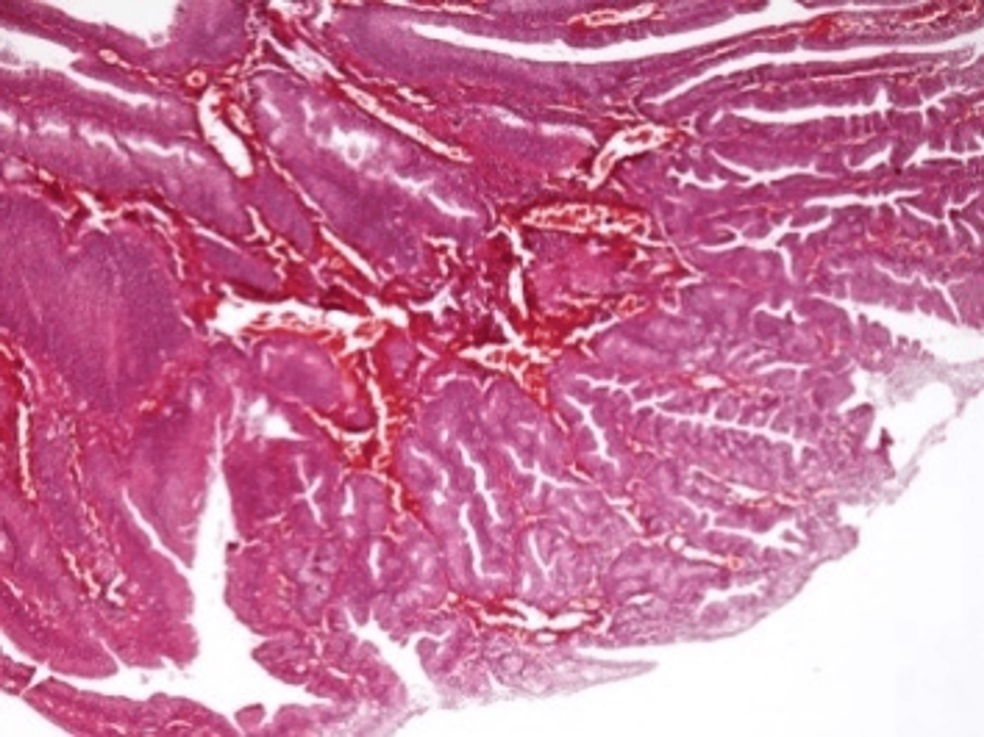
Histopathology: complex tumor architecture Microscopic evaluation of the tumor showing more complex architecture with irregular glands, without significant pleomorphism and no tumor invasion (hematoxylin and eosin stain, 100× magnification).

Microscopic examination revealed a villous adenoma characterized by multiple areas of severe dysplasia (Figures 4-6) and a single, small area of tumor invasion within the lamina propria. Immunohistochemical tests were performed for smooth muscle actin (SMA, a type of actin found in muscle cells), Ki67 (a marker for cell proliferation), adenomatous polyposis coli (APC, a tumor suppressor protein), MutL homolog 1 (MLH1, a DNA mismatch repair protein), postmeiotic segregation increased 2 (PMS2, another DNA mismatch repair protein), MutS homolog 2 (MSH2, also involved in DNA mismatch repair), and MutS homolog 6 (MSH6, a further DNA mismatch repair protein). These tests showed no invasion into the muscularis mucosa or submucosa (Figures 5-6).

**Figure 7:**
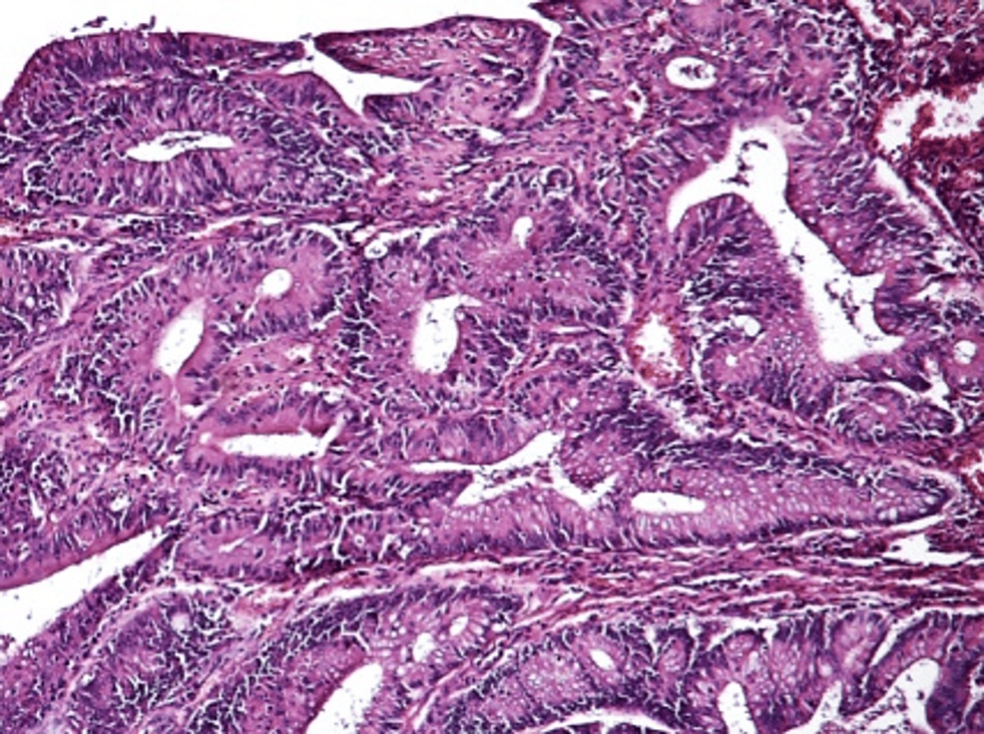
Histopathology: epithelial proliferation with moderate nuclear atypia Microscopic image showing epithelial proliferation of columnar cells with moderate nuclear atypia, displaying a glandular and alveolar pattern (hematoxylin and eosin stain, 20× magnification).

**Figure 8:**
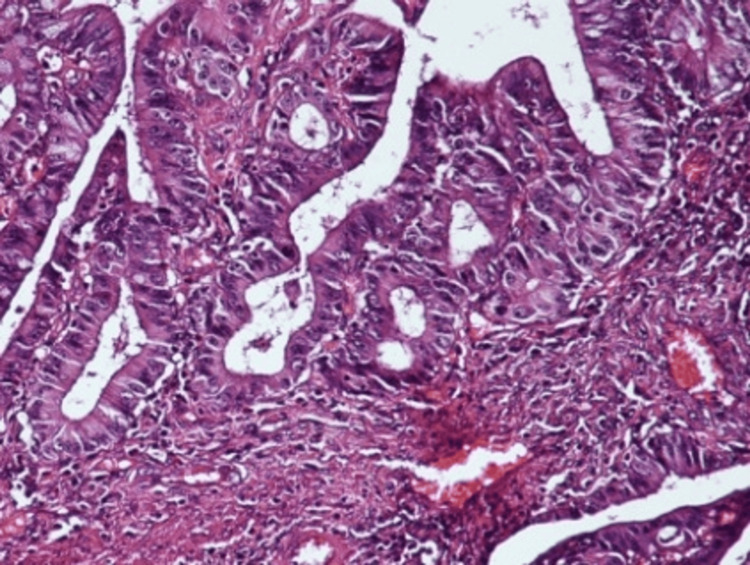
Histopathology: glandular architecture and stromal infiltration Microscopic view revealing complex glandular architecture and stromal infiltration by small clusters of atypical cells in an area with a desmoplastic stromal response, with no invasion into the muscularis mucosa or submucosa (hematoxylin and eosin stain, 20× magnification).

**Figure 9:**
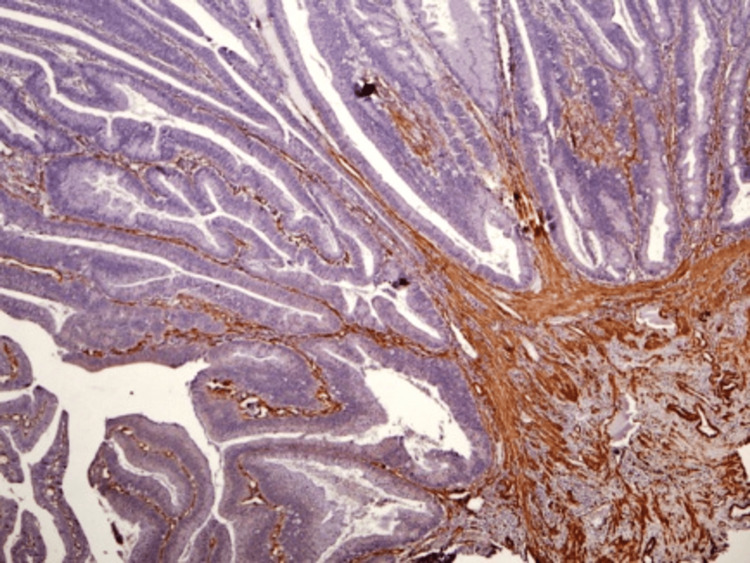
Histopathology: no muscularis mucosa invasion Microscopic evaluation showing no invasion of the muscularis mucosa (smooth muscle actin coloration, 100× magnification).

**Figure 10:**
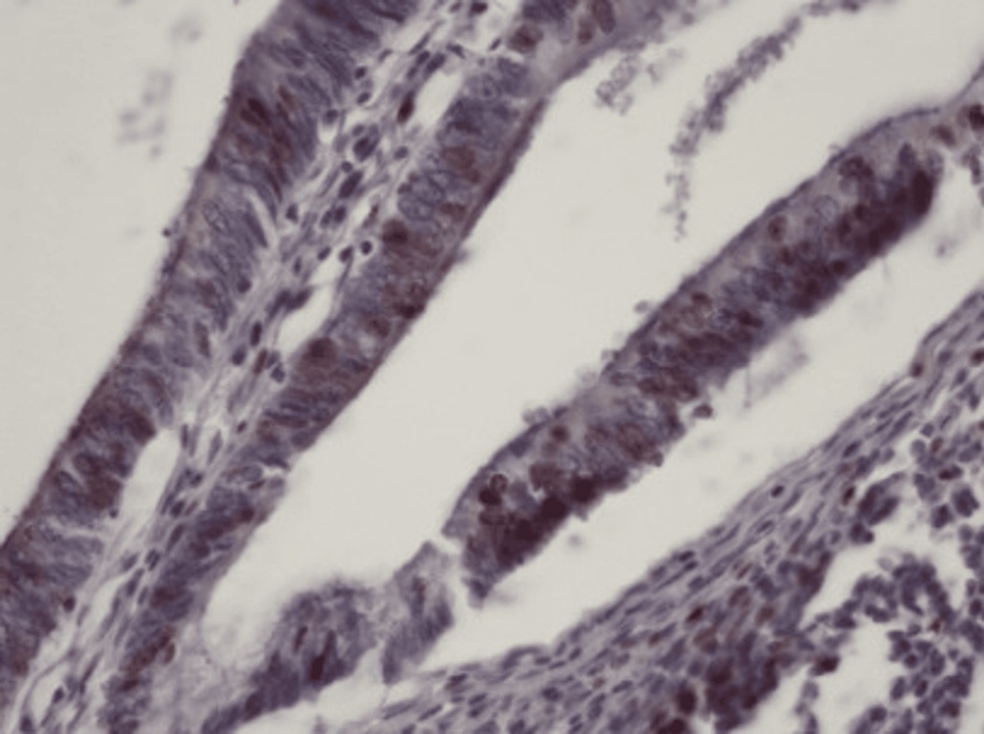
Immunohistochemistry: Ki67 cancer-marker evaluation Microscopic view with immunohistochemistry showing relatively rare tumor nuclei positive for the Ki67 cancer marker (200× magnification).

**Figure 11:**
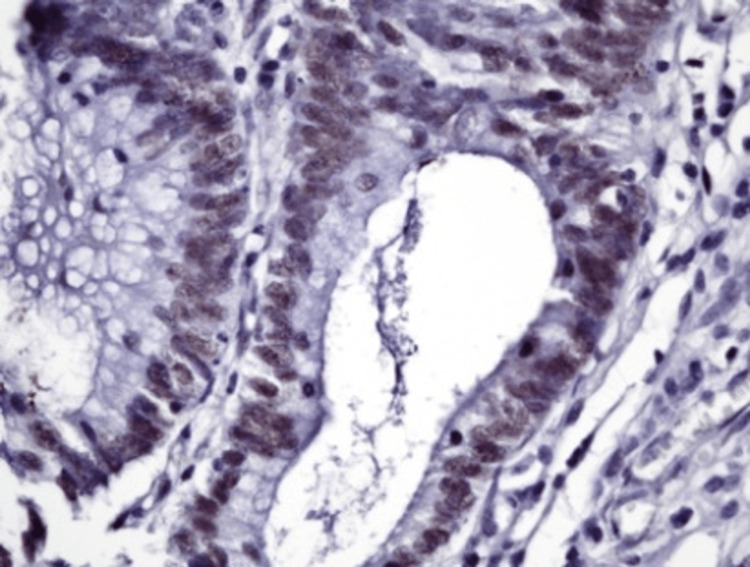
Immunohistochemistry: microsatellite instability markers evaluation Microscopic evaluation and immunohistochemistry showing tumor cells with various levels of positivity for microsatellite instability markers, indicating no microsatellite instability within the tumor.

**Figure 12:**
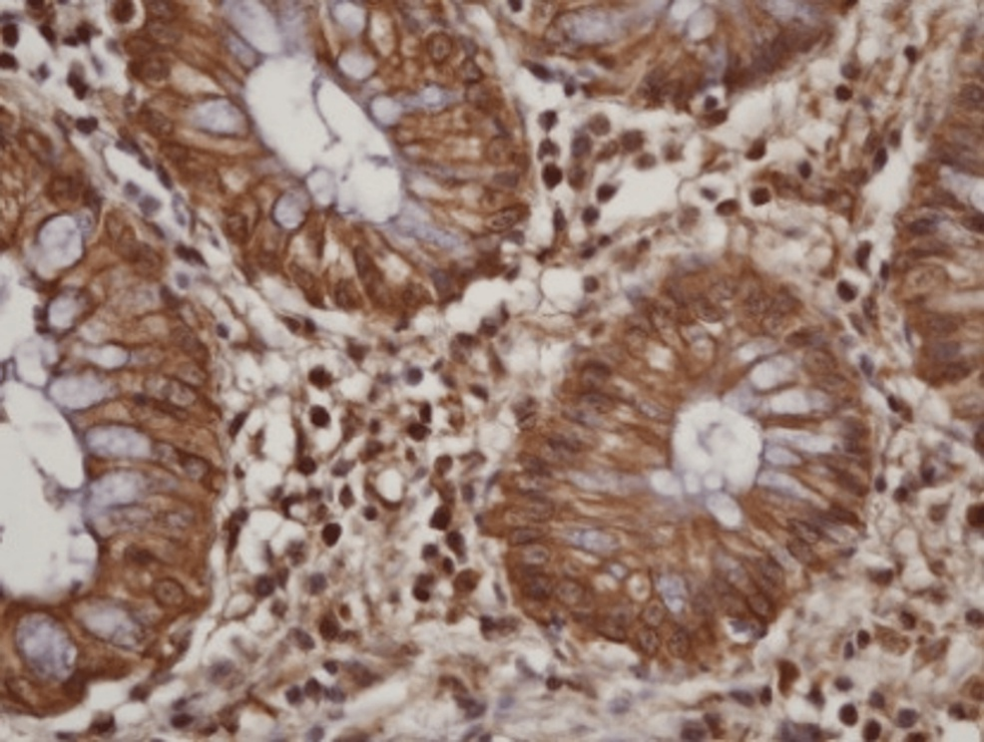
Immunohistochemistry: comprehensive microsatellite instability analysis Microscopic view with immunohistochemistry showing no microsatellite instability within the tumor. Tumor markers used include MutL homolog 1 (MLH1), postmeiotic segregation increased 2 (PMS2), MutS homolog 2 (MSH2), MutS homolog 6 (MSH6), and adenomatous polyposis coli (APC; 200× magnification).

The histopathological examination confirmed the diagnosis of a villous adenoma with high-grade epithelial neoplasia, which included high-grade dysplasia and in situ carcinoma (Figures 7-12). It also identified limited areas of well-differentiated tubular adenocarcinoma invading the lamina propria without clear evidence of submucosal invasion (pathologic tumor in situ).

## Discussion

Adenomatous polyps, especially those of significant size, have a high potential for malignant transformation. This risk escalates with the polyp's size [6]. Morson's research on 2,499 tubular and villous adenomas demonstrated 1.3%, 9.5%, and 46% malignant transformation rates in polyps smaller than 1 cm, 1-2 cm, and larger than 2 cm, respectively [7]. While most polyps found during colonoscopies are small and easily removable through endoscopic procedures, larger rectal polyps pose a significant challenge, particularly those over 5 cm or circumferentially developed [8]. These lesions can originate near the dentate line and extend to the middle or upper rectum. Despite their size, they may not undergo malignant transformation. Furthermore, imaging techniques like CT scans, magnetic resonance imaging, or double-contrast barium examinations are not always conclusive in detecting malignancy [9]. A colonoscopy offers a direct view of the rectum and the entire colon, allowing for multiple biopsies. However, it carries the risk of missing smaller lesions.

Rectal prolapse induced by a villous adenoma is a rare but serious complication, often associated with constipation and increased intra-abdominal pressure. Some studies suggest a higher malignancy incidence in such cases, citing constipation and colonic mucosal irritation as risk factors [10,11]. Others consider rectal prolapse to be a sign of malignancy [12]. Because approximately 90% of colorectal cancers originate from adenomatous polyps, endoscopic polypectomy has significantly reduced colorectal cancer incidence in developed countries [13].

Determining the appropriate treatment for these lesions requires a tailored approach for each case. Endoscopic polypectomy is the standard procedure for polyp removal, but its efficacy is limited in certain scenarios. Even advanced techniques like endoscopic mucosal resections or endoscopic submucosal dissections may not always yield the desired outcome, and approximately 2% to 10% of cases are unsuitable for endoscopic intervention. Challenges include postoperative complications like bleeding or perforation. For "carpet-like" adenomas or when malignancy cannot be ruled out, alternative treatments such as transanal excision and transanal endoscopic microsurgery are considered for limited, non-circumferential lesions [14]. More invasive procedures, like open colorectal resection or laparoscopic surgery, are necessary when less invasive methods are inadequate. In cases involving low rectal or anal canal tumors, abdominoperineal resection of the rectum with a permanent colostomy may be required. However, sphincter-preserving resections with subsequent anastomosis can be performed in some cases, yielding satisfactory results [15,16].

When there is uncertainty about the benign nature of a villous tumor, it is prudent to treat it as malignant [17]. Some studies report significant postoperative pathology discrepancies, with 50% of lesions reclassified as invasive cancer [18]. Biopsies in this context are often inaccurate, missing invasive cancer in approximately 40% of cases [18]. Complete excision is the recommended treatment, though entire rectum removal may be necessary [19]. Despite complete excision, recurrence rates can be as high as 40% [20]. Abdominoperineal resection is rarely necessary for benign rectal villous tumors but was employed in our case, where distinguishing between adenoma and cancer was challenging. This case also prompts a reevaluation of the pathological definitions of dysplasia, intraepithelial neoplasia, carcinoma in situ, and intramucosal adenocarcinoma in colorectal pathology.

## Conclusions

Giant rectal adenomas, while rare, can grow to substantial sizes without becoming malignant, leading to complications like prolapse, obstruction, hemorrhage, or hypersecretory symptoms. Preoperative diagnosis is difficult in cases of giant villous tumors, as large areas exhibiting histopathological features of villous adenoma with varying dysplasia degrees may coexist with invasive adenocarcinoma areas. The risk of overlooking a malignant lesion is significant, making surgical decision-making challenging. Although preoperative histopathology indicated a villous adenoma and conservative surgery was tempting, therapeutic decisions must prioritize patient welfare and the principles of beneficence and nonmaleficence. In cases involving giant, circumferential rectal lesions, conservative resection may not guarantee oncological safety or physiological functionality. Therefore, from a risk-benefit perspective, radical surgery, although potentially resulting in a permanent colostomy and altering the patient's quality of life, remains the safer alternative.
